# Salivary Metalloproteinase-8 and Metalloproteinase-9 Evaluation in Patients Undergoing Fixed Orthodontic Treatment before and after Periodontal Therapy

**DOI:** 10.3390/ijerph18041583

**Published:** 2021-02-08

**Authors:** Ioana-Andreea Sioustis, Maria-Alexandra Martu, Liana Aminov, Mariana Pavel, Petru Cianga, Diana Cristala Kappenberg-Nitescu, Ionut Luchian, Sorina Mihaela Solomon, Silvia Martu

**Affiliations:** 1 Periodontology Department, Faculty of Dentistry, “Grigore T. Popa” University of Medicine and Pharmacy of Iasi, 700115 Iași, Romania; ioana.sioustis@gmail.com (I.-A.S.); cristala.nitescu@gmail.com (D.C.K.-N.); ionut_luchian@yahoo.com (I.L.); drsolomonro@gmail.com (S.M.S.); parodontologie1@yahoo.com (S.M.); 2 Endodontics Department, Faculty of Dentistry, “Grigore T. Popa” University of Medicine and Pharmacy of Iasi, 700115 Iași, Romania; lianaaminov@yahoo.com; 3 Immumology Department, Faculty of Medicine, “Grigore T. Popa” University of Medicine and Pharmacy of Iasi and “St. Spiridon” Hospital of Iasi, 700115 Iași, Romania; mariana.pavel86@gmail.com (M.P.); petrucianga@hotmail.com (P.C.)

**Keywords:** biomarkers, inflammation, orthodontic treatment, periodontal treatment

## Abstract

(1) Background: Metalloproteinase-8 (MMP-8) and metalloproteinase-9 (MMP-9) are members of a family of proteases of major importance during orthodontic tooth movement. Their levels increase during orthodontic therapy and in periodontally affected tissues. Orthodontic fixed appliances retain dental plaque and can cause gingival inflammation. When gingival inflammation is present, the forces produced during orthodontic tooth movement can aggravate tissue reaction and cause the destruction of supportive periodontal tissue. This study aimed to identify biomarkers that facilitate the assessment of periodontal status during orthodontic treatment. (2) Methods: Our study was conducted on 111 patients who were about to receive fixed orthodontic treatment. We determined the salivary levels of MMP-8 and MMP-9 and bleeding on probing (BOP) before applying the orthodontic fixed appliance (T1), one week after appliance placement (T2), and during orthodontic treatment, one month after non-surgical periodontal treatment (T3). (3) Results: Patients undergoing orthodontic treatment show a significant increase in BOP, MMP-8, and MMP-9 levels one week after orthodontic appliance placement (T2) and a decrease in these parameters one month after periodontal treatment (T3). Statistically significant correlations were found between MMP-8 levels and BOP values at T1, T2, and T3. (4) Conclusion: In our study patients undergoing orthodontic treatment show a significant increase in BOP, MMP-8, and MMP-9 levels one week after orthodontic appliance placement and a decrease in these parameters one month after periodontal treatment. Strong positive statistically significant correlations were found between MMP-8 levels and BOP and medium positive statistically significant correlations between MMP-9 and BOP values before and after orthodontic treatment and periodontal treatment. MMP-8, MMP-9, and BOP could be used to assess the periodontal status of orthodontic patients.

## 1. Introduction

Orthodontic tooth movement (OTM) involves comprehensive periodontal and alveolar bone remodeling [1,2]. It is considerably different from physiological tooth movement [3] since it begins with an inflammatory-like response that involves the activation of different biological factors and degradation/synthesis of the extracellular matrix (ECM) in the periodontal ligament (PDL) [4]. The key trigger factor responsible for OTM is the pressure exerted on PDL cells and the extracellular matrix, which causes changes in the gene expression within cells and the extracellular matrix, and also induces the release of specific cytokines and chemokines. In response to mechanical loading, cytokines and chemokines control alveolar bone remodeling. Orthodontic forces induce capillary vasodilatation in the periodontal ligament, resulting in inflammatory cell migration and cytokine production [2].

Matrix metallo-proteinases (MMPs) are a family of proteases that are important in remodeling the extracellular matrix (ECM) [1]. A total of 23 human MMPs have been reported to date [5]. Among these, metalloproteinase-8 (MMP-8) and metalloproteinase-9 (MMP-9) are members of the collagenase and gelatinase groups, respectively [5]. They are initially synthesized as inactive proenzymes that may be stimulated in the ECM by proteolytic processing [5]. MMPs and TIMPs together play a major role in periodontium physiological remodeling [6] and the response to mechanical forces during orthodontic treatment [7]. MMP inhibition by synthetic MMP inhibitors has been shown to decrease OTM [8]. MMP-8 is deposited in an inactive form, specifically in granules of polymorphonuclear leukocytes (PMNs), and is mainly thought to be regulated by its selective granular release from triggered PMNs at inflammation sites [9]. Additionally, MMP-8 (human neutrophil collagenase, collagenase-2) is also released by certain non-PMN lineage cells, such as gingival fibroblasts, bone, and plasma cells [10]. MMP-8 is the most effective in hydrolyzing type I collagen [11] and is the primary interstitial collagenase in inflamed human gingiva [7].

A healthy periodontium is crucial to prevent any unsatisfactory changes to the tissues that support the teeth [12]. Pathogenic bacteria in close contact with the gingival margins are the key etiological agents for the development of periodontal disease [13]. Gingivitis is a periodontal disease that manifests without periodontal attachment loss; however, it exhibits a change in the equilibrium between the biofilm and the host. Gingivitis can progress to periodontitis, which is associated with attachment loss and bone loss [13]. Fixed orthodontic devices may increase supragingival biofilm accumulation and degrade periodontal health [12], increasing the amounts of pathogenic anaerobic bacteria in supra- or subgingival biofilms during orthodontic therapy. Therefore, proper hygiene is needed to prevent the development of gingivitis and periodontitis.

Although several biomarkers have been considered for the diagnosis of periodontal disease, there is no consensus regarding the biomarkers for monitoring bone resorption in orthodontic treatment. Bleeding on probing (BOP) can be readily evaluated and is useful for early diagnosis [14] and prevention of periodontal disease since it precedes other clinically detectable signs of gingivitis [15]. Furthermore, it correlates with the severity of inflammatory conditions in the gingival tissue [16]. If persistently present during the monitoring period, it represents a significant prognostic factor for periodontal impairment at the level of a particular situs. BOP sites exhibit a greater probability of severe attachment loss when compared to non-bleeding sites [17]. Substantial plaque accumulation and increased BOP are associated with orthodontic therapy [18]. Patients with high BOP are “at-risk” and demand a more rigorous periodontal therapy regimen than those with little to no BOP [19].

Since a majority of orthodontic patients will exhibit inflamed, swollen, bleeding gingiva at one point at least during treatment, suitable caution is required, and supportive periodontal care should be routinely recommended as an essential component of orthodontic therapy [19]. Reports have illustrated the value of a full-mouth examination at six sites per tooth for a detailed analysis of orthodontic patients’ periodontal status [20]. However, this approach commands a long and time-consuming clinical diagnosis that depends on the clinician’s expertise. Moreover, this process must be repeated at regular intervals to determine the patient’s periodontal status at recall visits. To the best of our knowledge, this is the first study to evaluate salivary biomarkers in patients undergoing orthodontic treatment before and after periodontal therapy.

This study aimed to evaluate the changes in the levels of matrix metalloproteinase-8 and matrix metalloproteinase-9 before and during orthodontic treatment and also after periodontal treatment and to analyze their correlation with the bleeding on probing index (BOP). Furthermore, we aimed to identify markers that could be used to investigate the periodontal status of orthodontic patients and to emphasize the need for regular periodontal maintenance during orthodontic treatment.

## 2. Materials and Methods

All the steps of this study were thoroughly explained to the patients prior to enrollment. The patients were instructed about the purpose of the study and provided informed consent before participating in the study. The study was conducted in accordance with the Helsinki Declaration. The protocol was approved by the Ethics Committee of the University of Medicine and Pharmacy from Iasi, Romania (Protocol identification code 29.01.2020/2540).

### 2.1. Subjects

This study was conducted on 111 patients aged between 18 and 39 years, with a mean age of 25.5 ± 5.4 years. All patients who were recruited completed the study. We included patients in generally good health who were about to receive fixed-appliance treatment and had a healthy periodontal status. The exclusion criteria were diagnosis of periodontal disease or a history of treatment, immune disease, systemic disease, smoking, pregnancy, lactation, and use of any medication that could interfere with OTM (antihistamines, cortisone, and hormones) within three months preceding the beginning of the study and use of antibiotics in the last six months.

All patients received oral hygiene instructions prior to the beginning of the study. We determined the BOP index and the levels of MMP-8 and MMP-9 before placing the orthodontic fixed appliance (T1), one week after appliance placement (T2), and during orthodontic treatment, one month after applying the periodontal non-surgical treatment (T3). Orthodontic treatment was performed with a Roth prescription 0.022-in bracket slot appliance, which was bonded to the maxillary or mandibular arch. The first archwire was a 0.012-in nickel-titanium conventional wire.

The periodontal treatment aimed to eliminate supragingival and subgingival plaque and calculus. This was accomplished by comprehensive scaling and professional brushing using ultrasonic instruments (Hu-Friedy, Symmetry IQ^®^ 3000, Chicago, IL, USA) and Gracey curettes (Hu-Friedy, Chicago, IL, USA).

The BOP score was evaluated as the proportion of bleeding sites (dichotomous yes/no evaluation) at six sites (mesiobuccal, buccal, distobuccal, mesiolingual, lingual, and distolingual) at the bottom of the sulcus/pocket on all present teeth when stimulated by a standardized manual periodontal probe [21]. A single examiner, blinded to the purpose of the study, performed all measurements during the clinical evaluation. For the intra-examiner calibration, 20 non-study individuals with orthodontic treatment were selected. The intra-examiner reproducibility was 90%. A periodontal specialist, different from the examiner performed the periodontal treatment during the study.

### 2.2. Saliva Sampling

Sample collection was performed during routine appointments. This procedure was performed before any other clinical procedure in order to avoid blood contamination. Unstimulated whole saliva (3–4 mL) was collected in the morning from all participants (the subjects were instructed to skip oral hygiene that morning), at approximately 10 AM “a jeune” by instructing the patients to passively drool in a sterile polypropylene tube which was immediately frozen in a dry ice bath and stored at −80 °C until biomarker assessment.

Unstimulated saliva was collected before placement of orthodontic appliances (T1), after the placement of orthodontic appliances but before periodontal therapy (T2), and during orthodontic treatment, one month after applying the periodontal treatment (T3).

Saliva samples were centrifuged at 13,000 rpm for 1 min at 4 °C to remove cellular and insoluble debris. The supernatant was then transferred in a new Eppendorf 1.5 mL tube and appropriately labeled. Following the pre-processing steps, all samples were kept at −80 °C until analysis.

The saliva samples were processed according to the manufacturer’s instructions. For MMP analyses, we used the Human MMP-8 (Matrix Metalloproteinase 8) ELISA Kit and Human MMP-9 (Matrix Metalloproteinase 9) ELISA Kit from Elabscience, China.

### 2.3. Statistical Analysis

Statistical analysis was conducted using SPSS 25.0 (SPSS^®^ version 25 for Windows^®^, SPSS Inc./IBM Group, Armonk, NY, USA) software, and *p* < 0.05 was considered to indicate a statistically significant difference.

The Kolmogorov-Smirnov test was used to test the normality of data (sample size > 50 respondents), which is a prerequisite for many statistical tests because normal data is an underlying assumption in parametric testing. The normality of the data for MMP-8 and MMP-9 levels and BOP values was tested separately for each of the three phases: T1 (before application of the orthodontic treatment), T2 (after application of orthodontic treatment), and T3 (after application of orthodontic and periodontal treatment). The null hypothesis for this test is that the data are normally distributed, and it was accepted (*p*-value > 0.05) for MMP-8 (T3), BOP% (T1), BOP% (T2), BOP% (T3). For double confirmation, a normal Q-Q plot was used to graphically visualize the normal distribution of the variables.

The Wilcoxon signed-rank test for nonparametric statistics was used to compare MMP-8 (T3) with MMP-8 (T2), MMP-9 (T3) with MMP-9 (T2), MMP-8 (T3) with MMP-8 (T1), MMP-9 (T3) with MMP-9 (T1), and to determine for each of these measurements (MMP-8 and MMP-9) if the values at T3 were significantly lower than those at T2 and significantly higher than those at T1.

The paired t-test (parametric test), the equivalent of the Wilcoxon Signed Test for parametric variables, was used to compare the BOP (T3) with BOP (T2) and the BOP (T3) with BOP (T1), and to determine if the values at T3 were significantly lower than those at T2 and significantly higher than those at T1.

Pearson and Spearman correlations were used to test whether there was a statistically significant linear relationship between MMP-8 levels and BOP values for all three stages: T1, T2, and T3. A significantly strong relationship was found between the two measurements at T2 (Spearman’s rho = 0.939, *p*-value < 0.001) and T3 (r = 0.842, *p*-value < 0.001) and a medium but statistically significant correlation was observed at T1 (Spearman’s rho = 0.614, *p*-value < 0.001). For a graphical visualization of this relationship, we used a scatter plot.

Spearman’s correlation was used to test whether there was a statistically significant linear relationship between MMP-8 and MMP-9 levels at all three stages: T1, T2, and T3. A significant and medium relationship was found between the two measurements at T3 (Spearman’s rho = 0.440, *p*-value < 0.01) and at phase T2 (Spearman’s rho = 0.239, *p*-value < 0.05). For a graphical visualization of this relationship, we used a scatter plot. The same type of correlation was used to test whether there is a statistically significant linear relationship between MMP-9 and BOP for all of the three stages: T1, T2, and T3. Two significant and medium relationship were found at phase T2 (Spearman’s rho = 0.314, *p* < 0.01) and T3 (Spearman’s rho = 0.426, *p* < 0.01).

Descriptive analyses were used to calculate descriptive coefficients such as mean, standard deviation, minimum, and maximum for all the variables included in the sample. The box plot was used to graphically visualize the difference between means and distribution for each of the three measurements (MMP-8, MMP-9, and BOP) within the three stages: T1, T2, and T3.

In order to determine the ROC (receiver operating characteristic) curve, we divided the patients into three groups: healthy group (BOP < 10%), localized gingivitis group (BOP ≥ 10% and BOP ≤ 30%), and generalized gingivitis group (BOP > 30%) [21] and calculated the cut-off point for MMP-8 and MMP-9 in all three stages: T1, T2, and T3.

## 3. Results

We analyzed the salivary MMP-8 and MMP-9 levels before orthodontic treatment (T1), one week after orthodontic appliance placement (T2), and during orthodontic treatment, one month after applying the periodontal treatment (T3), as described in the materials and methods. For salivary MMP-8 levels, the highest values were recorded at T2, with a mean value of 0.267 ± 0.20 ng/mL, while the lowest values were recorded at T1, with a mean value of 0.10 ± 0.07 ng/mL (Table 1).

**Figure 1:**
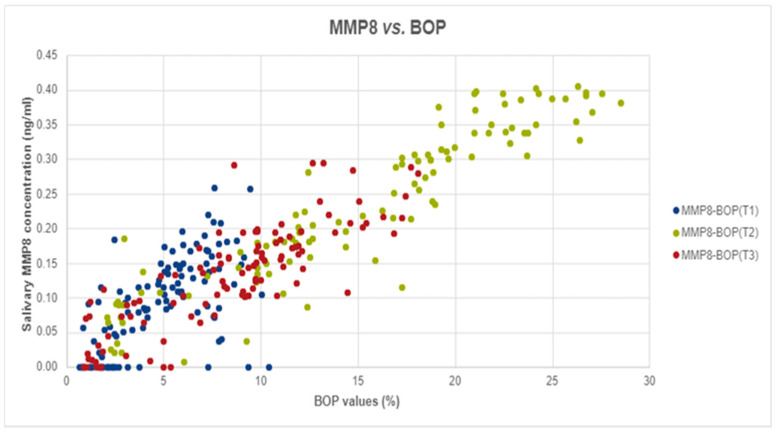
The correlation between salivary MMP-8 and BOP values in T1, T2, and T3.

After evaluating and comparing the values, we found that the mean value of MMP-8 at T3 was significantly lower than at T2 (*p*-value < 0.01) and significantly higher than that at T1 (*p*-value < 0.01) (Figure 1, Table 1).

**Figure 2:**
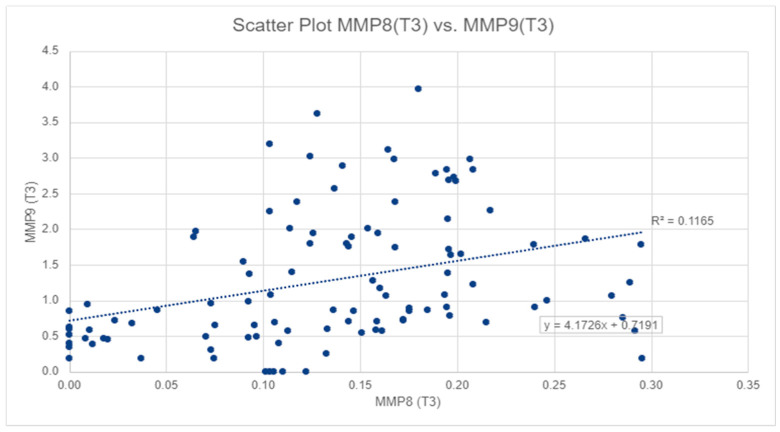
The correlation between salivary MMP-8 and MMP-9 values in T3.

For salivary MMP-9 levels, the highest values were also observed at T2, with a mean of 1.89 ± 1.82 ng/mL, and the lowest value was observed at T1, with a mean of 0.45 ± 0.48 ng/mL (Table 1), also in Figure 2, we can observe a similar pattern to MMP-8. The mean MMP-9 level at T3 was significantly lower than that at T2 (*p*-value < 0.01), but significantly higher than that at T1 (*p*-value < 0.01). However, salivary MMP-8 and MMP-9 levels displayed a significant moderate correlation at T3 (Spearman’s rho = 0.440, *p*-value < 0.01) and at T2 (Spearman’s rho = 0.239, *p*-value < 0.05) (Figure 2).

Similarly, the BOP values were assessed before placing the orthodontic fixed appliances (T1), one week after appliance placement (T2), and one month after periodontal treatment in this group of patients undergoing orthodontic treatment (T3). The highest BOP values were measured at T2, with a mean of 16.22% ± 8.84%, while the lowest BOP values were registered at T1, with a mean of 5.08% ± 2.72% (Table 1). The values of BOP at T2 were significantly higher than those at T3 (*p*-value < 0.01) and BOP values at T3 were significantly higher than those at T1 (*p*-value < 0.01) (Table 1).

Spearman Correlation analyses was performed to assess the potential correlation between MMP-8 levels and BOP. In our analyses we found strong, positive, and significant correlations at T2 (Spearman’s rho = 0.939, *p*-value < 0.001) and T3 (r = 0.842, *p*-value < 0.001) and medium, positive, and significant correlation at T1 (Spearman’s rho = 0.614, *p*-value < 0.001), as shown in Figure 1.

**Figure 3:**
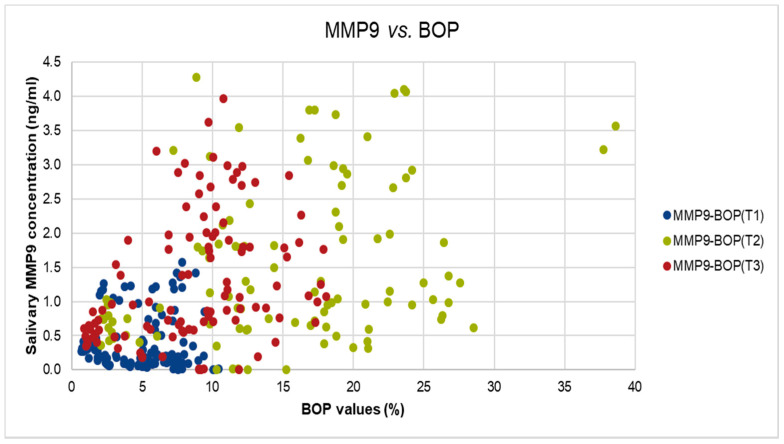
The correlation between salivary MMP-9 and BOP values in T1, T2, and T3.

We did identify also significant but moderate correlation between MMP-9 levels and BOP values at T2, and T3 (T2: Spearman’s rho = 0.314, *p*-value < 0.01, T3: Spearman’s rho = 0.426, *p*-value < 0.001), as shown in Figure 3.

As anticipated, compared to the healthy group measurements for all three sampling times (T1, T2, T3), the localized gingivitis group showed higher values for BOP, MMP-8, and MMP-9 compared with the healthy group and the generalized gingivitis group showed higher values for all three markers than the localized gingivitis group (Table 2).

Mann-Whitney U test was performed to investigate differences among the three groups, the localized gingivitis group showed significantly higher levels of MMP-8, MMP-9 compared with the healthy group, the same results also comparing the markers from the localized gingivitis group versus generalized gingivitis group.

**Figure 4:**
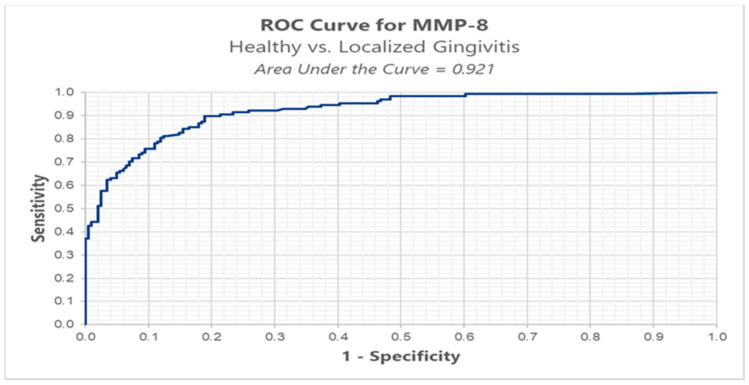
ROC analysis of MMP-8 in healthy versus localized gingivitis.

We conducted ROC analysis in order to determine a cut-off for MMP-8, between healthy (BOP < 10%) versus localized gingivitis group (BOP ≥ 10% and BOP ≤ 30%). The results highlight an optimal cut-off, using the Youden index method, of 0.152 ng/mL for which we have a sensitivity of 89.8% and a false positive of 18.9% (Table 3, Figure 4).

Results from ROC analysis of salivary biomarker levels of MMP-8 comparing the localized gingivitis group (BOP ≥ 10% and BOP ≤ 30%) to the generalized gingivitis (BOP > 30%) group and the healthy group (BOP < 10%) to the generalized gingivitis group (BOP > 30%) resulted in an optimal cut-off of 0.420 ng/mL, respectively 0.491 ng/mL. These results are not statistically significant because in the group of patients with generalized gingivitis there were only five patients.

**Figure 5:**
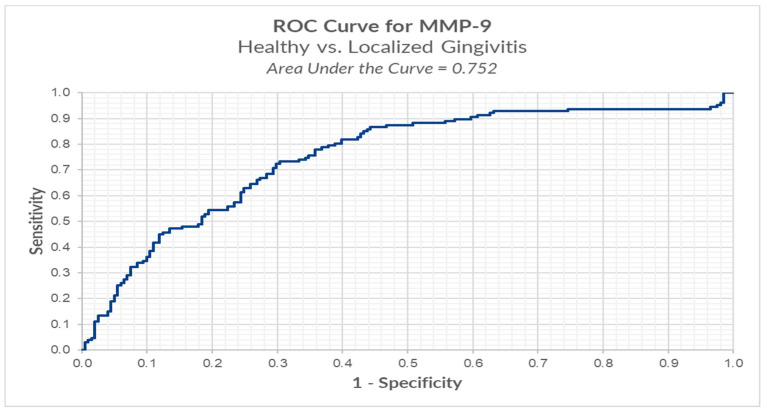
ROC analysis of MMP-9 in healthy versus localized gingivitis.

We conducted ROC analysis in order to determine a cut-off for MMP-9, between healthy versus localized gingivitis group. The results reveal an optimal cut-off using the Youden index method of 0.874 ng/mL (for MMP-9) for which we have a sensitivity of 73.2% and a false positive of 30.3% (Table 4, Figure 5).

Results from ROC analysis of salivary biomarker levels of MMP-9 comparing localized gingivitis group to generalized gingivitis group and healthy group to generalized gingivitis group resulted in an optimal cut of 0.491 ng/mL, respectively 2.923 ng/mL. These results are not statistically significant because in the group of patients with generalized gingivitis there were only five patients.

## 4. Discussion

The findings of this study suggest that patients undergoing orthodontic treatment show a significant increase in BOP and MMP-8 and MMP-9 levels one week after orthodontic appliance placement (T2) and a decrease in these parameters one month after periodontal treatment (T3). Statistically significant correlations were found between MMP-8 levels and BOP values at T1, T2, and T3. Metalloproteinase-8 levels increased in inflammatory status, since it is the primary interstitial collagenase under inflammatory conditions, as stated by Ingman [7]. This could explain the strong positive correlation found in our study between MMP-8 levels and BOP values.

Periodontal complications are one of the most frequent adverse effects of orthodontic treatment [22], and they include gingivitis, periodontitis, gingival recession or hypertrophy, alveolar bone loss, dehiscence, fenestrations, interdental folds, and dark triangles [23]. In addition, some researchers have shown clinical and microbiological changes in patients undergoing orthodontic treatment that partially normalize after the removal of the appliances [24]. The assumption that long-term fixed appliances may lead to undesirable, but inevitable, qualitative changes in subgingival bacterial biofilms that gradually become periodontopathogenic over time is illustrated by various studies [25,26].

Trombelli et al. [21] show that gingival inflammation can be properly and easily detected and assessed using BOP. Its absence is a good indicator for periodontal stability [27,28], so it has the ability to reflect the periodontal status and the severity of inflammation. Although useful for scientific purposes, the BOP approach presents some disadvantages [29], such as the amount of time necessary for the quantitative analysis and the difficulty in distinguishing differences in the evaluation scale during a regular, thorough periodontal examination [30]. As other studies have shown, BOP is used to evaluate the results of preventive and treatment strategies for periodontal diseases [31]. Nonetheless, oral fluid biomarkers exhibit the ability to provide further, more accurate insight when compared to regular clinical investigations [32]. Moreover, those investigations (BOP, plaque index, probing depth, clinical attachment level, and radiographic recordings [33]) illustrate only retrospective data, and not the current disease status [33,34].

In light of these factors, identification of a specific biomarker for assessing periodontal status during OTM is important. This is necessary since completion of orthodontic treatment without effects on the periodontium is essential but challenging. One should also consider the frequent iatrogenic effects caused by orthodontic treatment; some authors agree that preventive measures must be considered for all patients undergoing orthodontic therapy [35].

Various studies have shown that increased MMP-8 and MMP-9 levels characterize not only periodontal disease [36,37] but also tend to increase during OTM [4]. Our study evaluated MMP-8 and MMP-9 levels and BOP at T1, T2, and T3 and identified a significant positive correlation between the MMP-8 levels and BOP before and after periodontal treatment. Indeed, these findings are in agreement with the results of other studies in which MMP-8 levels were highly correlated with BOP [38,39]. Furthermore, we observed a medium positive statistically significant correlation between MMP-9 and BOP values before and after orthodontic treatment and periodontal treatment.

In our study, we conducted ROC analysis in order to determine a cut-off for MMP-8 and MMP-9 between healthy versus localized gingivitis group versus generalized gingivitis. Results from the ROC analysis of salivary biomarker levels of MMP-8 and MMP-9 comparing healthy versus localized gingivitis resulted in an optimal cut-off of 0.152 ng/mL and respectively 0.874 ng/mL. This is the first study to analyze such a value in orthodontic patients and we believe it is a valuable tool that can assess the current periodontal status and prognosis of a patient and can be further studied in patients with more severe periodontal disease.

Thus, we propose the use of MMP-9 and especially MMP-8 levels as biomarkers of periodontal disease during orthodontic treatment to facilitate the detection of early periodontitis or gingivitis. Although BOP and MMP-8 levels have been shown to allow distinction between a healthy periodontal status and gingivitis or periodontitis cases [38,40], other studies have shown conflicting or contrary results [41,42]. MMP-8 is associated with the diagnosis of periodontal disease [43], the severity of periodontal inflammation, evolution, and follow-up of therapy [38,40,44]. It can also be used to monitor periodontal disease status [40]. Therefore, these biomarkers can be used to identify the inflammatory status of patients undergoing orthodontic treatment and to measure results after periodontal treatment.

The major component of the periodontal extracellular matrix is collagen type I. MMP-8 levels have been shown to be correlated with collagen type I degradation products, overcoming the protective mechanism of MMP tissue inhibitors in active disease sites as opposed to inactive sites in patients with periodontitis and healthy controls [37]. MMP-8 is the key collagenolytic component found in the gingival tissue and oral fluids [45]. Therefore, MMP-8 is considered a biomarker in periodontitis. This could explain its significant and strong correlation with BOP. A recent study by Shirozaki et al. [46] found that the percentage of sites with BOP increased after orthodontic therapy, as our data also confirms.

In our study, salivary MMP-8 levels in patients undergoing both orthodontic and periodontal treatment were 0.5-fold smaller than those before applying periodontal treatment, which is in agreement with the study performed on the gingival crevicular fluid of patients with no orthodontic appliances by Mäntylä et al. [47]. Interestingly, Marcaccini et al. [48] found strong correlations between the plasma levels of MMP-8 and MMP-9 before and after periodontal treatment in patients without orthodontic appliances. Thus, further studies with larger groups of cases might clarify any potential links among MMP-8, MMP-9, and BOP before periodontal treatment (in the current study with only 111 subjects, the *p*-value for the correlation between MMP-9 and BOP was <0.01). Since we only aimed to evaluate the local inflammation status using biomarkers such as salivary MMP-8 and MMP-9 values and BOP percentages, we conceived the study without including any other clinical measurements.

This is the first study to evaluate salivary biomarkers in patients undergoing orthodontic treatment before and after periodontal therapy. A biomarker is easy to assess, takes less chair time, and documents the current inflammatory status. Here, we propose that the MMP-8 level combined with BOP values could be analyzed as a biomarker before and during orthodontic treatment in order to identify the individual periodontal inflammatory status and disease prognosis.

Nevertheless, the study had some limitations. The sample size was small, and evaluation of data after three, six, and 12 months or at each month during the first six months would have yielded more applicable results. Future studies could include an assessment of each patient’s measures of hygiene (by means of questionnaires or by plaque index evaluations) in order to identify more specific correlations between results and the used hygiene methods.

## 5. Conclusions

In our study patients undergoing orthodontic treatment show a significant increase in BOP, MMP-8, and MMP-9 levels one week after orthodontic appliance placement and a decrease in these parameters one month after periodontal treatment. Strong positive statistically significant correlations were found between MMP-8 levels and BOP and medium positive statistically significant correlations between MMP-9 and BOP values before and after orthodontic treatment and periodontal treatment. MMP-8, MMP-9, and BOP could be used to assess the periodontal status of orthodontic patients.

## Figures and Tables

**Table 1:** Summarized levels of Metalloproteinase-8 (MMP-8), metalloproteinase-9 (MMP-9), and bleeding on probing (BOP) before orthodontic treatment (T1), one week after orthodontic appliance placement (T2), and during orthodontic treatment, one month after applying the periodontal treatment (T3).

Parameter	Mean (±Standard Deviation)
MMP9(T1)	0.450 ± (0.48) ng/mL
MMP9(T2)	1.899 ± (1.82) ng/mL
MMP9(T3)	0.100 ± (0.07) ng/mL *#
MMP8(T1)	0.100 ± (0.07) ng/mL
MMP8(T2)	0.267 ± (0.20) ng/mL
MMP8(T3)	0.140 ± (0.08) ng/mL *#
BOP(T1)	5.088 ± (2.72)%
BOP(T2)	16.224 ± (8.84)%
BOP(T3)	8.761 ± (4.56)% **##

T1—before orthodontic treatment; T2—one week after orthodontic appliance placement; T3—one month after combined orthodontic-periodontal treatment; BOP—bleeding on probing; MMP8—matrix metalloproteinase-8; MMP9—matrix metalloproteinase-9. *: significant difference compared to T2 (*: *p* < 0.01), using Wilcoxon Signed Ranks Test. #: significant different compared to T1 (#: *p* < 0.01), using Wilcoxon Signed Ranks Test. **: significant difference compared to T2 (**: *p* < 0.01), using Paired Sample *T*-test. ##: significant different compared to T1 (##: *p* < 0.01), using Paired Sample *T*-test.

**Table 2:** Characteristics of study measurements among healthy, localized gingivitis and generalized gingivitis groups.

Parameter	Healthy Group (N = 201)	Localized Gingivitis Group (N = 127)	Generalized Gingivitis Group (N = 5)
MMP-9 (ng/mL)	0.843 ± 1.11	1.842 ± 1.58 **	3.532 ± 0.50 ##**
MMP-8 (ng/mL)	0.098 ± 0.06	0.249 ± 0.09 **	0.994 ± 0.26 ##**
Bleeding on probing (BOP, %)	5.259 ± 2.80	16.411 ± 5.06 **	39.366 ± 1.17 ##**

BOP—bleeding on probing; MMP-8—matrix metalloproteinase-8; MMP-9—matrix metalloproteinase-9. *: significant different compared to healthy group (**: *p* < 0.01). #: significant different compared to gingivitis group (##: *p* < 0.01).

**Table 3:** Results from ROC analysis of individual salivary biomarker levels comparing healthy group to localized gingivitis group.

Group	Optimal Cut-Off	Sensitivity	Specificity	FP	FN	AUC
MMP-8 (Healthy versus Localized Gingivitis)	0.152 ng/mL	0.898	0.811	0.189	0.102	0.924

MMP-8—matrix metalloproteinase-8.

**Table 4:** Results from ROC analysis of individual salivary biomarker levels of MMP-9 comparing the healthy group to the localized gingivitis group.

Group	Optimal Cut-Off	Sensitivity	Specificity	FP	FN	AUC
MMP-9 (Healthy versus Localized Gingivitis)	0.874 ng/mL	0.732	0.697	0.303	0.268	0.752

MMP-9—matrix metalloproteinase-9.

## Data Availability

The data used to support the findings of this study are available from the correspondence author upon request.
